# Erratum to: Procedures and recommended times in the care process of the patient with pancreatic cancer: PAN-TIME consensus between scientific societies

**DOI:** 10.1007/s12094-017-1636-z

**Published:** 2017-03-15

**Authors:** R. Vera, A. Ferrández, C. J. Ferrer, C. Flores, C. Joaquín, S. López, T. Martín, E. Martín, M. Marzo, A. Sarrión, E. Vaquero, A. Zapatero, J. Aparicio

**Affiliations:** 1 Spanish Society of Medical Oncology, Madrid, Spain; 2 Spanish Society of Pathological Anatomy, Madrid, Spain; 3 Spanish Society of Radiation Oncology, Madrid, Spain; 4 Spanish Society of General and Family Physicians, Madrid, Spain; 5 Spanish Society of Endocrinology and Nutrition, Madrid, Spain; 6 Spanish Society of Surgical Oncology, Madrid, Spain; 7 Spanish Society of Medical Radiology/Spanish Society of Abdominal Radiology, Madrid, Spain; 8 Spanish Association of Surgeons, Madrid, Spain; 9 Spanish Society of Family and Community Medicine, Madrid, Spain; 10 Spanish Society of Primary Care Physicians, Madrid, Spain; 11 Spanish Association of Gastroenterology, Madrid, Spain; 12 Spanish Society of Internal Medicine, Madrid, Spain

## Erratum to: Clin Transl Oncol DOI 10.1007/s12094-016-1609-7

The article *Procedures and recommended times in the care process of the patient with pancreatic cancer: PAN*-*TIME consensus between scientific societies*, written by R. Vera, A. Ferrández, C. J. Ferrer, C. Flores, C. Joaquín, S. López, T. Martín, E. Martín, M. Marzo, A. Sarrión, E. Vaquero, A. Zapatero, J. Aparicio, was originally published electronically on the publisher’s internet portal (currently SpringerLink) on 19 January 2017 without open access.

With the author(s)’ decision to opt for Open Choice the copyright of the article changed on 15 March to © The Author(s) 2017 and the article is forthwith distributed under the terms of the Creative Commons Attribution 4.0 International License (http://creativecommons.org/licenses/by/4.0/), which permits use, duplication, adaptation, distribution and reproduction in any medium or format, as long as you give appropriate credit to the original author(s) and the source, provide a link to the Creative Commons license and indicate if changes were made.

